# Medical Reports of the Effects of Arsenic in the Cure of Agues, Remitting Fevers, and Periodic Head-Achs

**Published:** 1786

**Authors:** Thomas Fowler

**Affiliations:** Physician to the General Infirmary of the County of Stafford


XV. *Medical Reports of the Effects of Arſenic in the Cure of Agues, Remitting Fevers, and periodic Head-Achs. By* Thomas Fowler, *M. D. Phyſician to the General Infirmary of the County of Stafford. Together with a Letter from Dr.* Arnold, *of Leiceſter, and another from Dr.* Withering, *deſcribing their Experience of the Effects of Arſenic in the Cure of Intermittents.* 8vo. Johnſon, *London*, 1786.

FOR more than a century paſt arſenic has been occaſionally recommended by different writers in the cure of intermittents; but the generality of phyſicians have hitherto been unwilling to adopt ſo hazardous and ſuſpicious a remedy in a diſeaſe which is not often fatal, and for which we have a ſpecific of ſo much certainty and ſafety as the Peruvian bark; to ſay nothing of a variety of other remedies, which are often ſucceſsfully adminiſtered, either ſeparately, or combined with the bark, in ſuch fuch cafes. — It is probable too, that the large and dangerous dofes, of three, four, and even five grains *, in which it appears formerly to have been adminiftered, may have deterred them from making ufe of a medicine which, in fuch dofes, could hardly fail to produce the moft alarming effects.

Wepfer, who had been inftructed in the ufe of an arfenical decoction of great efficacy in fevers, did not think it prudent to communicate the manner of preparing it to the public †; and Lentilius, who mentions the great fuccefs of a phyfician at Coppingen with a fimilar preparation, which is defcribed as infallible in every fpecies of intermittent, the quartan not excepted ‡, very cautioufly adds, that he him-

* Thefe dofes we find mentioned by different writers; but in the Mifcell, Curiof. (Dec. 2, ann. 5.) we meet with the formula of a fever powder, each dofe of which contains only about a quarter of a grain of arfenic. Even this, however, muft be confidered as a dangerous dofe.

† Nollem illam publici juris fieri, ne imperitis aut temerariis nocendi anfam darem, eoque magis cum tot febrifuga tutiora et efficaciora habeantur. — Wepfer de Cicuta Aquatica, cap. 20, hift. 13, fchol. 3.

‡ Tollit omnes intermittentes, ipfamque adeo quartanam. — Mifc. Cur. Acad. Nat. Cur. Dec. 2, ann. 3. Obf. 46. 4to. Norimbergæ, 1685.

felf felf preferred a flower, but fafer method of cure *. Friccius, however, who had repeatedly experienced the efficacy of arfenic, and who was a zealous friend to the medical ufe of poifons in general, in a learned and curious work †, publifhed at the beginning of the prefent century, not only afferts, that, in the cure of intermittents, the effects of arfenic are as certain as thofe of the Peruvian ‡ bark, but contends that it poffeffes all the properties which are wont to be afcribed to the beft remedies, and that it never deceives the expectation either of

* Ego alia methodo intermittentes feliciter abigere didici, tardiori licet, atqui tutiori; nolim hanc imitari.—Mifc. Cur. Dec. 2, ann. 3. Obf. 46.

† Melchioris Friccii, Medici Ulmenfis, Tractatus medicus de virtute venenorum medica; in quo paradoxologice et contra communem medicorum opinionem experimentis, rationibus, et celeberrimorum in arte medica virorum authoritatibus probatur, venena interne et externe ufurpata non effe noxia, fed præftantiffima remedia, et in morbis defperatis, ut lue venereâ, leprâ, maniâ, epilepfia, febribus quartanis, &c. ultimum medicorum et ægrotorum refugium, et eorum correctiones et præparationes ut plurimum effe fuperfluas et inutiles, fimulque cujufque veneni antidotum oftenditur. 8vo. Ulmæ, 1701.

‡ Paroxyfmi ab ejus (arfenici) ufu tam certo profligati, quam certo decantatiffimum corticem Chin-de China, alias Peruvianum appellatum, eos profligare, quotidie experimur.—Friccius, de virt. venen. med. Cap. *de Arfenico.*

the the phyſician or patient *. Notwithſtanding this eulogium, however, and the praiſes beſtowed on it about the ſame time by Slevogtius †, who has mentioned his ſucceſsful adminiſtration of it in fifty caſes, its uſe for many years paſt, as a remedy for fevers, ſeems to have been confined almoſt entirely to empirical practice.—Newman, in his chemiſtry, ſpeaks of a ſpecific febrifuge, as it was called, which was ſold at Berlin and other places, and was ſaid to be a preparation of arſenic; and in this country a medicine of a ſimilar nature has for ſeveral years paſt been advertiſed under the title of the taſteleſs ague and fever drops. Theſe drops having been ſucceſsfully employed in the coun-

* Experientia nos docebit arſenicum in febribus intermittentibus exhibitum omnes eas dotes poſſidere, quibus optima remedia prædita eſſe debent. Non enim ſolum cito effectum ſuum in morbum exſerit, una vel altera exhibitione ſtatim paroxiſmum febrilem protelando, non ſolum tuto operatur, nullum periculoſum ſymptoma excitando, vel morboſam corpori indiſpoſitionem relinquendo; neque ſolum jucunde curat, quia in reſtrictiſſima doſi ſumitur, omnis odoris et ſaporis moleſti expers eſt, atque ideo citra nauſeam ingeritur; ſed etiam et quod maximum, remedium eſt certum, et quod deſiderium ægrotantis, votumque medici nunquam fallit.—Ibid. p. 36.

† Propempticon Inaugurale de permiſſione prohibitorum et prohibitione permiſſorum.—4to. Jena, 1700.

ty ty infirmary at Stafford, and found by Mr. Hughes, the apothecary to the infirmary, to be a preparation of arsenic, Dr. Fowler dissolved a small portion of white arsenic in a solution of the fixed vegetable alkali, and, on trying and comparing the effects of this solution with those of the patent ague drops, he found the two medicines to be similar, but that the former was too strong: he therefore diluted the solution by doubling the proportion of water, and then comparing the effects of the two medicines, found them to be nearly of equal strength.

Having thus become acquainted with what he esteemed a powerful medicine, he administered it in a great number of cases, and now communicates his experiments and observations on this subject to the public, relating, with great candour, all the instances of failure, as well as success, that have occurred to him in the course of its administration.

The medicine employed by the author is prepared by dissolving sixty-four grains of white arsenic, finely powdered, and the same quantity of a pure fixed vegetable alkali (or, instead of the alkali, a double proportion of purified nitre) in half a pint of common distilled water, in in a proper veſſel, placed in a ſand bath, the water being ſuffered to boil gently till the ſolution is complete. To this ſolution, when cold, are added half an ounce of compound ſpirit of lavender, and a ſufficient quantity of common diſtilled water, to make the exact weight of the ſolution fifteen ounces and a half Troy weight.

Eighty drops (or one drachm) of the ſolution, thus prepared, contain about half a grain of arſenic; and Dr. Fowler has adminiſtered it to adults with conſiderable latitude, from ten drops twice a day to more than double that number thrice a day: but although large doſes of it, exhibited three times a day, and with ſhort intermiſſions, are found to be more efficacious than ſmall ones given twice a day, and with long intervals, yet he very prudently gives the preference to moderate doſes, as theſe, he obſerves, are ſufficiently ſucceſsful, and their effects by no means ſo troubleſome and diſtreſſing to the patient as thoſe of larger ones. Twelve drops Dr. Fowler conſiders as a medium doſe for adults; but he does not confine its uſe to this period of life, having adminiſtered it with ſucceſs to children under two years of age. In general, however, he obſerves, it ought not to be had recourſe to at ſo early an age, unleſs the failure of other medicines, or the circumſtance of its being taſteleſs, and therefore more eaſily adminiſtered, ſhould render its uſe adviſeable; but, even in ſuch caſes, he cautions us not to perſiſt in it, if its effects become troubleſome.—The doſe for children, from two to four years old, varies from two to five drops; but, for the ſake of a more accurate meaſurement of the doſes for children under five years of age, we are directed to add an ounce of common diſtilled water to an ounce of the ſolution, in order to form a milder preparation, of which ten drops (being equivalent to five of the former) will be a doſe for a boy of five years old.

The ſolution, when given in ſmall doſes, will, in general, we are told, be attended with no operation. Sometimes, however, it will occaſion nauſea and griping; but theſe effects, the author obſerves, ſeldom increaſe to vomiting or purging, unleſs large doſes of the medicine are employed; and, he aſſures us, they may frequently be obviated, and the adminiſtration of the ſolution ſucceſsfully continued, by the aſſiſtance of ſmall doſes of laudanum.

Certain ſwellings, eſpecially of the face, or a loſs of appetite, have been obſerved by the author author sometimes to attend the larger doses of the solution, and now and then even the smaller ones.

In several instances, he observes, it has proved diuretic, while, in two or three others, it has seemed to diminish the natural urinary discharge. In a few cases it has occasioned an uneasiness and pain at the stomach, or a slight general eruption like the nettle rash; and in a very few instances he has seen it produce a sweat, a head-ach, or slight tremors. These effects, however, whether separately or collectively considered, have been so few and contingent, that they ought not, he thinks, to be deemed any part of the ordinary operation of the medicine.

The cases in which Dr. Fowler has administered the solution, and of which he gives an account, are divided into sections, in order that the cases which resemble each other in their nature, treatment, or event, and also the observations more immediately connected with each particular part of the subject, may appear in a more clear and practical point of view.

The first section contains fifty-two cases of agues, the greater part of them tertians, successfully treated by the solution. In these cafes, the author's ufual method is to give the folution three times a day, in a dofe fuited to the age of the patient, for five days; at the end of which time, if the fits, as, he affures us, moft commonly happens, are fufpended, he omits the ufe of the medicine for two or three days, and then repeats it three days more, in order to prevent a relapfe. This is his general direction relative to the ufe of the folution in the treatment of agues;—for the exceptions to it, which different circumftances may require, we muft refer our readers to the work itfelf.

The fecond fection contains twelve cafes of agues, which, after failure by the folution, were cured by the affiftance of the bark.

In the third fection are related fix cafes of agues, in which the folution failed to cure the difeafe. In all of thefe cafes the failure is afcribed to the irregular attendance of the patients.

The fourth and fifth fections contain fixteen cafes of remitting fevers, and feven of periodic head achs, in which the folution is faid to have been productive of the fame good effect as in agues.

The

The cafes which are the fubjects of the firft, fecond, and third fections, are only a part of the inftances of agues in which the author has made trial of the folution; but to fhew that they have been collected as a juft fpecimen of the practice he recommends, he has given, in the form of a table, a view of the curative effects of the folution in all the cafes of agues in which he has tried it. From this table it appears, that, of two hundred and forty fuch cafes, one hundred and feventy-one were completely cured by the folution; that forty-five were cured by the Peruvian bark, after failure by the folution; and that in twenty-four of the cafes it proved unfuccefsful, which the author attributes to a partial adminiftration of the medicine, occafioned by the irregular attendance of the patient.

The letters to the author from Dr. Arnold and Dr. Withering contain additional proofs of the efficacy of the folution in the cure of intermittents. The former of thefe phyficians, as we learn from his letter, has tried it in about eighty fuch cafes, quotidians, tertians, and quartans, and has feldom known it fail of fuccefs. In a few inftances he has feen reafon to lay it afide, and to truft the cure to the red bark, bark, which has ſucceeded; but the ſolution has, he believes, ſucceeded in as many inſtances, when the red bark has failed; and in no caſe has he been ſenſible that it produced any permanent ill effect. From Dr. Withering's letter we find, that, of forty-eight patients, thirty-three were cured by the uſe of the ſolution alone; three complained of pain in the ſtomach, loſs of appetite, and had ſwollen faces; but their fevers, it ſeems, were cured, and a little ſoluble tartar removed the ſymptoms now mentioned: the other twelve, we are told, received no benefit. Dr. Withering is convinced, that, in caſes where great debility has prevailed, either from old age or other cauſes, and where the recurrence of the fever, under the quotidian form, with the long protraction of the paroxyſms, and the great irritability of the ſtomach, have not allowed a ſufficient quantity of bark to be given, he has ſeen the exiſtence of the patients preſerved by the uſe of arſenic. In the courſe of his letter, Dr. Withering mentions, that Mr. Freer, jun. ſurgeon at Birmingham, has given it to more than a thouſand patients, without either hazard or inconvenience. — It may be proper to obſerve, that, in the ſolution employed by Dr. Arnold and

Dr.

Dr. Withering in their experiments, the proportion of arfenic was fmaller than in Dr. Fowler's folution, and that the medium dofe for an adult, ufed by Dr. Arnold, was equal only to about nine, and that employed by Dr. Withering to feven drops of Dr. Fowler's medicine.

Of the effects of this medicine, we can fay nothing from our own experience; but a judicious and experienced practitioner, who, on the authority of M. le Febure *, tried its effects in cancers, and who has likewife adminiftered it in agues and fome other difeafes, affures us, that, in many of the cafes, though given in fo minute a dofe as the fixteenth part of a grain, its effects were fo troublefome, that he was obliged to difcontinue it; and that he was never able to extend the dofe of it, in any cafe, beyond a quarter of a grain.

Sir George Baker, in his valuable obfervations on the late intermittent fevers †, informs us, that a medicine, compounded of arfenic and opium, (the dofe of which was a very few

* Remede approuvé pour guerir radicalement le Cancer occulte et manifefte ou ulcéré. 8vo. Paris, 1774.

† Medical Tranfactions, Vol. III. page 157.

drops drops in water) was taken by ſome of the inferior ranks of people, and ſometimes ſucceſsfully; but that, now and then, violent vomitings, cholic, and dyſentery, were the effects of it, eſpecially when a patient, too deſirous of a compendious cure, exceeded the doſe limited. The learned writer adds, that a practitioner in the county of Dorſet, who bought a large quantity of this medicine, and diſpenſed it among his patients, obſerved, that it hardly ever failed to ſtop the fits very ſoon; but that the fever was apt to return, though it was as eaſily prevented by the ſame means repeated. He had reaſon to think, however, that it injured the conſtitution afterwards in ſeveral inſtances; ſo that he was, at length, deterred from the uſe of it; and, we are told, he ſaw more than one caſe where an ague, thus cured, was followed by a palſy of the lower extremities.

While we thus bring forward to the notice of our readers circumſtances that may tend to render them cautious in the uſe of ſo active a ſubſtance, we think it right to obſerve, that Dr. Fowler, who contends only for its employment in what he conceives, from his experience, to be ſafe doſes, acknowledges that it is is a medicine of too great activity to be safely administered by those who are unacquainted with the practice of physic: but, we fear, that, even in the hands of medical practitioners, arsenic, generally adopted as a substitute for the bark in the cure of intermittents, would be productive of infinite mischief; and that a remedy which is capable of exciting virulent effects in such very minute doses, and which is frequently (as we learn from Dr. Fowler's work) rendered insupportable to the patient by the variation of only the sixtieth or eightieth part of a grain, would, in many cases, be worse than the disease.

